# Coexisting pulsion and traction diverticula revealed during planned Zenker peroral endoscopic myotomy

**DOI:** 10.1055/a-2974-6089

**Published:** 2026-10-13

**Authors:** Andrés Gutiérrez, Reem Al Shabeeb, Mustafa Naveed, William Abel, Karin Fleischer, Klaus Mönkemüller

**Affiliations:** 1 Unidad Académica Gastroenterología – Prof. Dra. Carolina OlanoHospital de Clínicas – Dr. Manuel QuintelaMontevideoUruguay; 2 Carilion School of Medicine – Department of Gastroenterology 1757 Virginia TechRoanokeVirginiaUnited States; 3 Department of GastroenterologyAMEOS Teaching University Hospital, Otto von Guericke University MagdeburgHalberstadtHalberstadtGermany

A 74-year-old woman with progressive dysphagia 16 years after C5–C7 anterior cervical discectomy and fusion (ACDF) was referred for Zenker peroral endoscopic myotomy because preprocedural esophagography had been interpreted as showing a Zenker diverticulum. At the start of the planned procedure, endoscopy showed a proximal pulsion-type Zenker diverticulum with an intervening septum. Further examination unexpectedly revealed a second, distinct pharyngoesophageal pouch containing exposed cervical fixation hardware ( Fig. 1 and Video 1 ). The procedure was aborted.

**Fig. 1:**
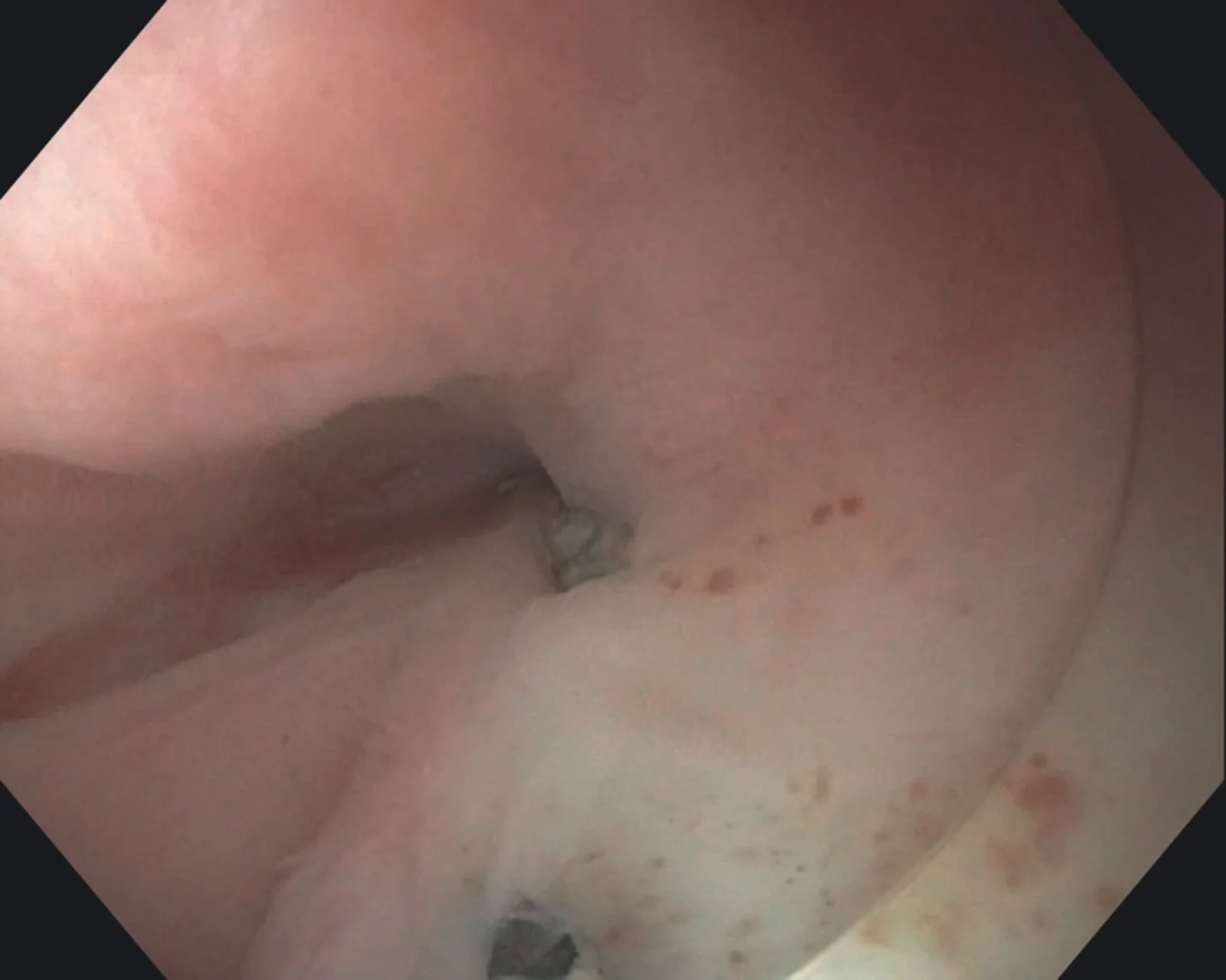
An endoscopic view of the second pharyngoesophageal pouch with exposed cervical fixation hardware, consistent with a post-ACDF traction diverticulum.

**Video 1** Planned Zenker peroral endoscopic myotomy reveals a Zenker diverticulum and a separate post-ACDF traction diverticulum containing exposed cervical fixation hardware.


These findings supported the coexistence of Zenker diverticulum and a post-ACDF traction diverticulum. A percutaneous endoscopic gastrostomy tube was placed for nutritional support, followed by the surgical removal of the cervical plate and screws. An esophageal wall defect adjacent to the hardware was repaired primarily and reinforced with a sternocleidomastoid muscle flap. Dysphagia improved substantially, although residual symptoms persisted. The remaining Zenker diverticulum was later treated with rigid endoscopic CO _2_ laser septotomy and cricopharyngeal myotomy. Postprocedural clinical follow-up after this later treatment was not available.

Zenker diverticulum is a pulsion pseudodiverticulum associated with impaired upper esophageal sphincter opening and cricopharyngeal dysfunction. 1​ In contrast, post-ACDF pharyngoesophageal diverticula are uncommon traction lesions associated with fibrosis, adherence, and chronic mechanical interaction between the pharyngoesophageal wall and the anterior cervical hardware. 2​ ​3​ They may present years after surgery and be associated with hardware exposure or esophageal wall injury. 2​ ​3​ In patients with previous ACDF, every cervical pouch should be examined for a relationship to fixation hardware before septotomy. Identification of a second pouch should prompt the termination of endoscopic therapy and multidisciplinary evaluation.

Endoscopy_UCTN_Code_CCL_1AB_2AC_3AF

## Data Availability

Data sharing is not applicable to this article, as no data sets were generated or analyzed during the current study.

## Declaration of GenAI Use

OpenAI Codex was used to assist with English-language editing, document structuring, reference formatting, and technical assembly of author-provided visual materials. No clinical images or video contents were generated by artificial intelligence (AI). The authors reviewed and verified all AI-assisted output and take full responsibility for the final content.
